# 830. Central Nervous System Involvement in Disseminated *Mycobacterium avium* Complex Infection in Patients with Newly Diagnosed HIV

**DOI:** 10.1093/ofid/ofab466.1026

**Published:** 2021-12-04

**Authors:** Antonio Camiro, Andrea Llamas-López, Mercedes Aranda-Audelo, David H Martínez-Oliva, Estefania Sienra-Iracheta, Patricia Rodriguez-Zulueta

**Affiliations:** 1 Centro Médico ABC, Mexico City, Distrito Federal, Mexico; 2 Hospital General Dr. Manuel Gea González, Mexico City, Distrito Federal, Mexico

## Abstract

**Background:**

Disseminated Mycobacterium avium complex (MAC) infection occurs in 20-40% of patients with < 50 CD4/mm^3^. Data describing central nervous MAC involvement (CNS-MAC) in disseminated infection is scarce.

**Methods:**

We conducted a retrospective case series in the outpatient infectious diseases clinic in the hospital “Dr. Manuel Gea Gonzales” in Mexico City. We reviewed all records from October 2020 to May 2021 and identified all culture proven MAC infections.

**Results:**

We found 7 cases of MAC, with disseminated infection (positive bone marrow cultures) with 3 out of those 7 meeting our definition for CNS-MAC (positive cerebrospinal fluid culture). All cases of CNS-MAC infection occurred in patients with < 50 CD4/mm^3^ and recent HIV diagnosis (1-4 months) that were referred to our institution with consumptive syndrome and fevers. All patients were receiving antiretroviral treatment (ART) with BIC/FTC/TAF and initiated ART in less than 1 month since HIV diagnosis. Opportunistic infections were ruled-out at the moment of CNS-MAC diagnosis (criptococcal meningitis, cytomegalovirus retinitis, tuberculosis and histoplasmosis). All patients exhibited non-specific neurologic symptoms at arrival (headache and bradipsiquia) mixed with more severe symptoms (one case of ataxia, one case of vertigo, one case of III nerve palsy). All patients were treated with Clarithromycin/Levofloxacin/Ethambutol. Two patients achieved symptom remission and 1 patient was lost to follow-up. Of important note, all CSF analysis and CNS imaging studies carried-out were normal. No MAC bacilli were identified with direct Ziel-Neelsen staining of CSF.

**Conclusion:**

We found a high proportion of CNS-MAC in patients with disseminated MAC infection (42.8%) during the study period. All patients presented CNS symptoms and normal CSF characteristics. In our setting, patients with suspected disseminated MAC infection CD4 counts < 50 cells/mm^3^ might represent a specific population that could benefit from routine targeted diagnostic test at presentation in order to establish CNS involvement.

**Disclosures:**

**All Authors**: No reported disclosures

